# A Triterpenoid Inhibited Hormone-Induced Adipocyte Differentiation and Alleviated Dexamethasone-Induced Insulin Resistance in 3T3-L1 adipocytes

**DOI:** 10.1007/s13659-015-0063-5

**Published:** 2015-06-16

**Authors:** Ji-Huan Qin, Jun-Zeng Ma, Xing-Wei Yang, Ying-Jie Hu, Juan Zhou, Lin-Chun Fu, Ru-Hua Tian, Shan Liu, Gang Xu, Xiao-Ling Shen

**Affiliations:** Laboratory of Chinese Herbal Drug Discovery, Tropical Medicine Institute, Guangzhou University of Chinese Medicine, Guangzhou, 510405 People’s Republic of China; State Key Laboratory of Phytochemistry and Plant Resources in West China, Kunming Institute of Botany, Chinese Academy of Sciences, Kunming, 650201 People’s Republic of China

**Keywords:** 6*α*-Hydroxylup-20(29)-en-3-on-28-oic acid, 3T3-L1, Adipocyte differentiation, Dexamethasone-induced insulin resistance, Adipocyte dysfunction, PI3K/Akt2 signaling

## Abstract

**Abstract:**

6*α*-Hydroxylup-20(29)-en-3-on-28-oic acid (**1**), a natural triterpenoid, was found to possess the ability in a dose-dependent manner inhibiting hormone-induced adipocyte differentiation in 3T3-L1 preadipocytes, and restoring glucose consuming ability in dexamethasone (DXM)-induced insulin resistant 3T3-L1 adipocytes. Compound **1** was also found to ameliorate DXM-induced adipocyte dysfunction in lipolysis and adipokine secretion. Mechanistic studies revealed that **1** inhibited adipocyte differentiation in 3T3-L1 preadipocytes via down-regulating hormone-stimulated gene transcription of peroxisome proliferator-activated receptor γ and CCAAT-enhancer-binding protein alpha which are key factors in lipogenesis, and restored DXM-impaired glucose consuming ability in differentiated 3T3-L1 adipocytes via repairing insulin signaling pathway and activating down-stream signaling transduction by phosphorylation of signaling molecules PI3K/p85, Akt2 and AS160, thus leading to increased translocation of glucose transporter type 4 and transportation of glucose.

**Graphical Abstract:**

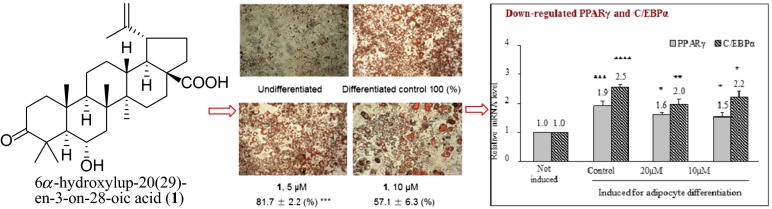

## Introduction

Obesity and obesity-induced insulin resistance increases the likelihood of major diseases referred as metabolic syndrome, such as heart disease, type 2 diabetes, stroke, etc. [1, 2]. Hypertrophic adipocytes in obesity release excess lipolytic products into blood, resulting in pathological changes and functional damage to organ tissues including pancreas, and inactivation of insulin receptor (IR), thus increase the risk of insulin resistance and type 2 diabetes [3, 4]. In addition, adipose tissue in obesity secrets elevated level of proinflammatory adipokines such as leptin, tumor necrosis factor-α (TNF-α) and interleukins (ILs) but lowered level of anti-inflammatory cytokine such as adiponectin, resulting in metabolic disorder and chronic inflammation of the body [5, 6]. Abnormally secreted adipokines may also reduce insulin sensitivity of target tissues by impairing insulin signaling transduction [6, 7]. Insulin resistance is characterized by reduced glucose uptake by target tissues or cells (adipose, muscle, liver) owing to impaired insulin signaling transduction and/or glucose transporting ability. In adipocyte, inactivation of insulin signaling molecules such as IR, insulin receptor substrate (IRS), PI3K and Akt, especially the down-stream PI3K and Akt, reduces the translocation of intracellular glucose transporter 4 (GLUT4) to plasma membrane and consequently impairs the glucose transporting ability of the cell [8]. Chinese herbal medicines contribute greatly to the research and development of new drugs. Structurally diverse compounds with bioactivities in reducing blood lipid and glucose level, or prevention of cardiovascular and metabolic diseases have been discovered, such as stilbenoids from *Polygonum multiflorum* and flavonoids from *Crataegus pinnatifida* [9, 10]. In our attempt to search natural components with hypoglycemic or hypolipidemic potential, 6*α*-hydroxylup-20(29)-en-3-on-28-oic acid (**1**) - a lupane type triterpenoid from *Viburnum odoratissimum*—was found to be able to enhance glucose uptake in dexamethasone-induced insulin resistant 3T3-L1 adipocytes and HepG2 cells [11]. Further investigations were then conducted and here we give a detail report for the effects and working mechanisms of **1** on hormone-stimulated adipogenesis, and dexamethasone-induced insulin resistance and adipocyte dysfunction. Rosiglitazone, which is an insulin sensitizer working as agonist of peroxisome proliferator-activated receptor γ, was used as the drug control.

## Results and Discussion

### Compound **1** Inhibited “Hormone Cocktail”-Induced Adipocyte Differentiation and Lipogenesis in 3T3-L1 Preadipocytes

**Fig. 1:**
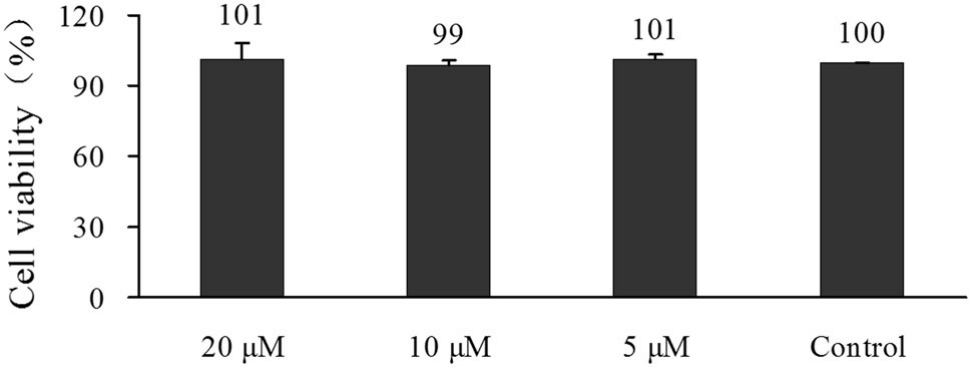
Compound **1** did not affect the proliferation of 3T3-L1 preadipocytes. 3T3-L1 preadipocytes were incubated with different concentrations of **1** for 48 h. Cell viability was measured by CCK-8 assay. Data were expressed as Mean ± SD of three independent experiments

**Fig. 2:**
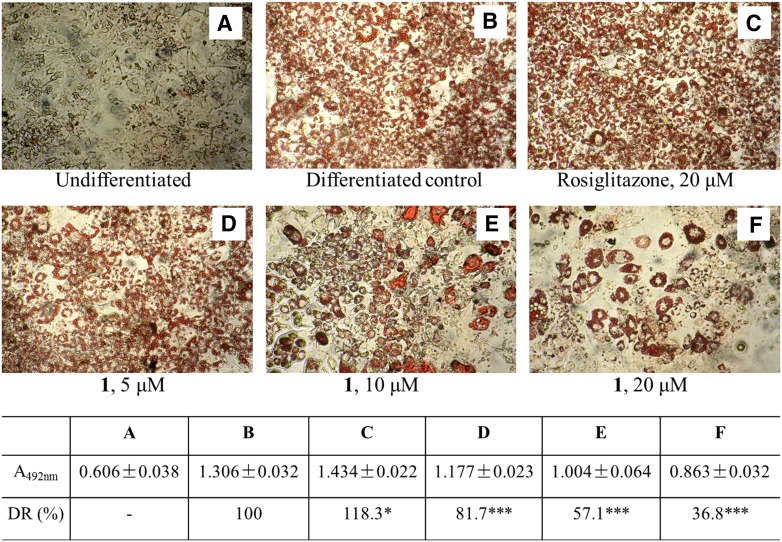
Detection of differentiated 3T3-L1 adipocytes. 3T3-L1 Preadipocytes were maintained in growth medium (Undifferentiated), or induced for adipocyte differentiation in the absence (Differentiated control) or presence of various concentrations of **1** or rosiglitazone. Differentiated adipocytes were stained red by oil red O. Pictures were taken under an inverted microscope (×100). Absorption of Oil red O for each treatment was measured at 492 nm (A_492nm_) after dissolving the cellular Oil red O with isopropanol, differentiation rate (DR) was calculated by equation DR (%) = 100 × (A_Sample_ − A_Undifferentiated_)/(A_Differentiated control_ − A_Undifferentiated_). *P < 0.05, ***P < 0.001 versus Differentiated control

In an 48-h proliferation assay, **1** at 20, 10 or 5 μM did not affect the viability of 3T3-L1 preadipocytes (Fig. 1), but in “hormone cocktail”-induced adipocyte differentiation, **1** dose-dependently reduced the staining of cells by Oil red O, indicating inhibited adipocyte differentiation. Differentiation ratio of 3T3-L1 preadipocytes in the presence of 20, 10 or 5 μM of **1** was remarkably decreased from 100 % of the control to 36.8, 57.1 or 81.7 % (*P* < 0.001 each), respectively (Fig. 2). In this experiment, rosiglitazone at 20 μM enhanced adipocyte differentiation as expected.

**Fig. 3:**
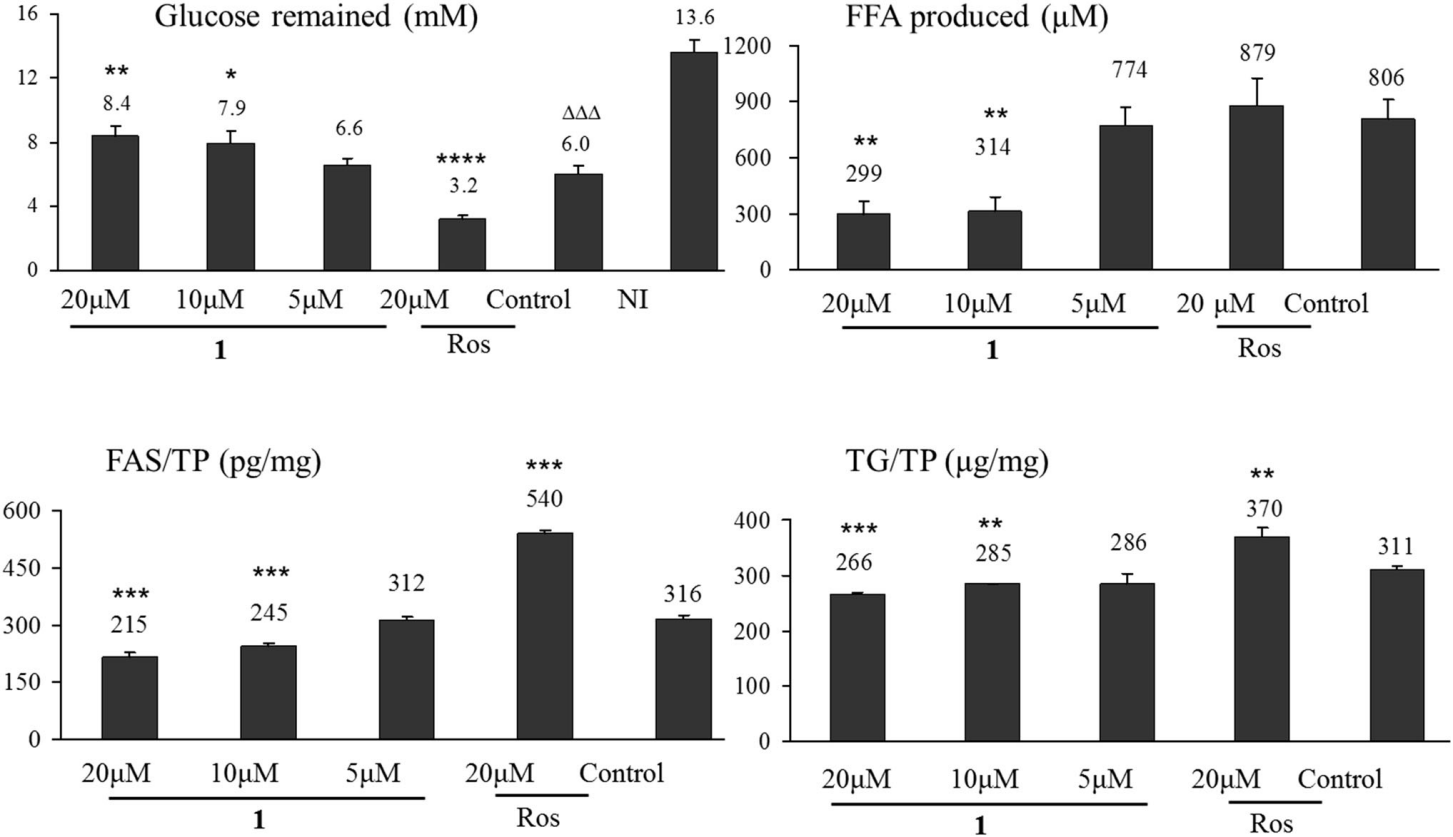
Compound **1** reduced glucose utilization and lipogenesis of 3T3-L1 cells in adipocyte differentiation. 3T3-L1 preadipocytes were induced for adipocyte differentiation for 8 days. Drug treatment was performed from day 1 to 4. Medium was replaced every other day. Glucose and FFA concentrations in medium on day 6 and cellular TG and FAS contents on day 8 were measured by using the kits. Cellular TG and FAS contents were normalized using total protein (TP). Data were expressed as Mean ± SD of three independent experiments. ^*^ *P* < 0.05, ^**^ *P* < 0.01 and ^***^ *P* < 0.001 versus Control. ^ΔΔΔ^ *P* < 0.001 versus Not induced. Ros: rosiglitazone

Triglyceride (TG) is the indicator of lipogenesis in fat cell. Synthesis of TG by cultural fat cells relies on the decomposition of glucose to generate free fatty acids (FFAs). Glucose utilization and FFA production are therefore indirect indicators for lipogenesis. As shown in Fig. 3, glucose remaining in culture medium of 3T3-L1 cells with differentiation induction was much less than that in medium of cells without induction (6.0 mM vs. 13.6 mM, *P* < 0.001), implying stimulated glucose utilization and lipogenesis in adipocyte differentiation.Unlike rosiglitazone which stimulated the glucose utilization and TG synthesis,  **1** at 20 or 10 μM increased the glucose remaining (*P* < 0.01 or 0.05), as a result, reduced FFA production (*P* < 0.01 each) and cellular TG content (*P* < 0.001 or 0.01) of induced cells, showing inhibitory effect on “hormone cocktail”-induced adipocyte differentiation and lipogenesis.

### Compound **1** Inhibited “Hormone Cocktail”-Stimulated Gene Transcription of Peroxisome Proliferator-Activated Receptor γ (PPARγ) and CCAAT-Enhancer-Binding Protein Alpha (C/EBPα)PPARγ and C/EBPα

**Fig. 4:**
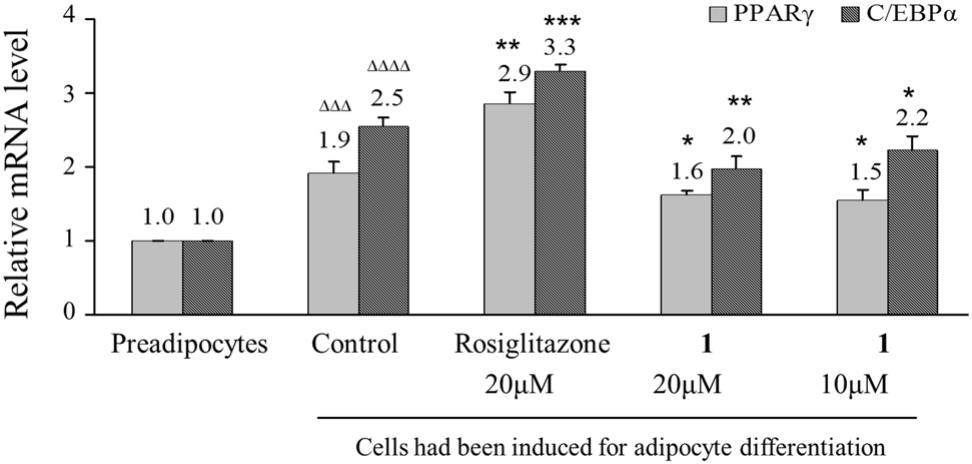
Compound **1** down-regulated PPARγ and C/EBPα gene transcription in 3T3-L1 cells in the process of adipocyte differentiation. 3T3-L1 preadipocytes were maintained in growth medium (Preadipocytes) or induced for adipocyte differentiation in the absence (Control) or presence of **1** or rosiglitazone for 8 days. mRNA levels were analyzed by RT-PCR, and normalized using β-actin. ^ΔΔΔ^ *P* < 0.001 and ^ΔΔΔΔ^ *P* < 0.0001 versus Preadipocytes. ^*^ *P* < 0.05, ^**^ *P* < 0.01 and ^***^ *P* < 0.001 versus Control

PPARγ and C/EBPα are two key transcriptional factors in adipocyte differentiation and lipogenesis, both express in very low level in preadipocytes but are significantly up-regulated in the process of adipocyte differentiation [12]. In this study, gene transcription of PPARγ and C/EBPα in 3T3-L1 cells were remarkably stimulated by “hormone cocktail” (*P* < 0.001 and 0.0001 vs. cells not induced), the addition of rosiglitazone (a PPARγ agonist) further stimulated the expression of the two genes, but the addition of **1** (20 or 10 μM) into the differentiation medium caused marked reduction in cellular mRNA levels of PPARγ (*P* < 0.05 vs. induced cell control) and C/EBPα *(P* < 0.01 or 0.05 vs. induced cell control) (Fig. 4). As a result, fatty acid synthase (FAS), the key enzyme catalyzing the synthesis of FFA [13], was suppressed by **1** (*P* < 0.001) (Fig. 3). In this study, **1** inhibited hormone-stimulated adipocyte differentiation and lipogenesis through down-regulating gene expression of PPARγ and C/EBPα, showing potential to deal with obesity and obesity-related health problems.

### Compound **1** Restored Glucose Consuming Ability and Alleviated Dysfunction in Dexamethasone-Induced Insulin Resistant 3T3-L1 Adipocytes

As mentioned above, obesity is a primary cause for development of insulin resistance, and insulin resistance always accompanies with metabolic disorder. In this study, effect of **1** on improvement of glucose consuming ability and metabolic disorder was investigated in insulin resistant 3T3-L1 adipocytes. Dexamethasone (DXM), which impairs glucose transporting ability and causes insulin resistance and metabolic disorder in adipose tissue and adipocytes [14, 15], was used to induced insulin resistant state of the cells.

**Fig. 5:**
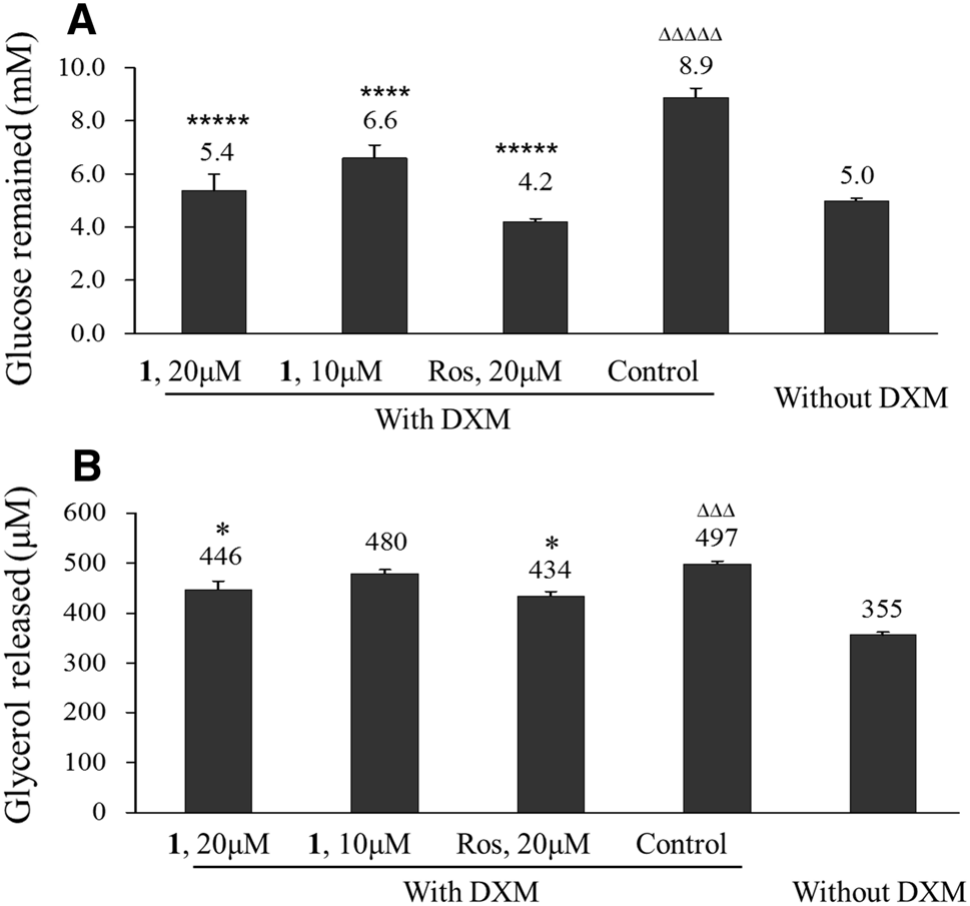
Compound **1** restored glucose consuming ability in DXM-treated 3T3-L1 adipocytes and inhibited DXM-stimulated lypolysis. Fully differentiated 3T3-L1 adipocytes were incubated with **1** or rosiglitazone (Ros) in the presence of 1 μM DXM for 48 h. Glucose and glycerol concentrations in medium were measured by using the assay kits. Data were mean ± SD of three repeated experiments. *P < 0.05, ^****^ *P* < 10^−4^ and ^*****^ *P* < 10^−5^ versus DXM-treated cells (Control); ^ΔΔΔ^ *P* < 10^−3^ and ^ΔΔΔΔΔ^ *P* < 10^−5^ versus DXM-free cells (Without DXM)

Reduced glucose uptake is the character of insulin resistance. In our study, 3T3-L1 adipocytes in the presence of 1 μM DXM consumed much less glucose within 48 h than cells in the absence of DXM (*P* < 10^−5^), indicating that insulin resistant state in 3T3-L1 adipocytes was successfully induced by DXM treatment. **1** at 20 and 10 μM enhanced the glucose consuming ability of DXM-treated 3T3-L1 adipocytes in a dose-dependent way (*P* < 10^−5^ and 10^−4^) like rosiglitazone did, implying that **1** restored the glucose transport ability of the cells (Fig. 5a).

Oversupplied lipolytic products contribute to insulin resistance. In this study, effect of **1** on lipolysis in DXM-treated 3T3-L1 adipocytes was monitored by measuring the glycerol concentration in culture medium. Result showed that, 3T3-L1 adipocytes in the presence of 1 μM DXM released much more glycerol into medium within 48 h than cells in the absence of DXM (*P* < 0.001), indicating stimulated lipolysis by DXM. Compound **1** and rosiglitazone at 20 μM reduced the glycerol concentration in medium (*P* < 0.05) (Fig. 5b), showing inhibitory effect on DXM-stimulated lipolysis in 3T3-L1 adipocytes.

**Fig. 6:**
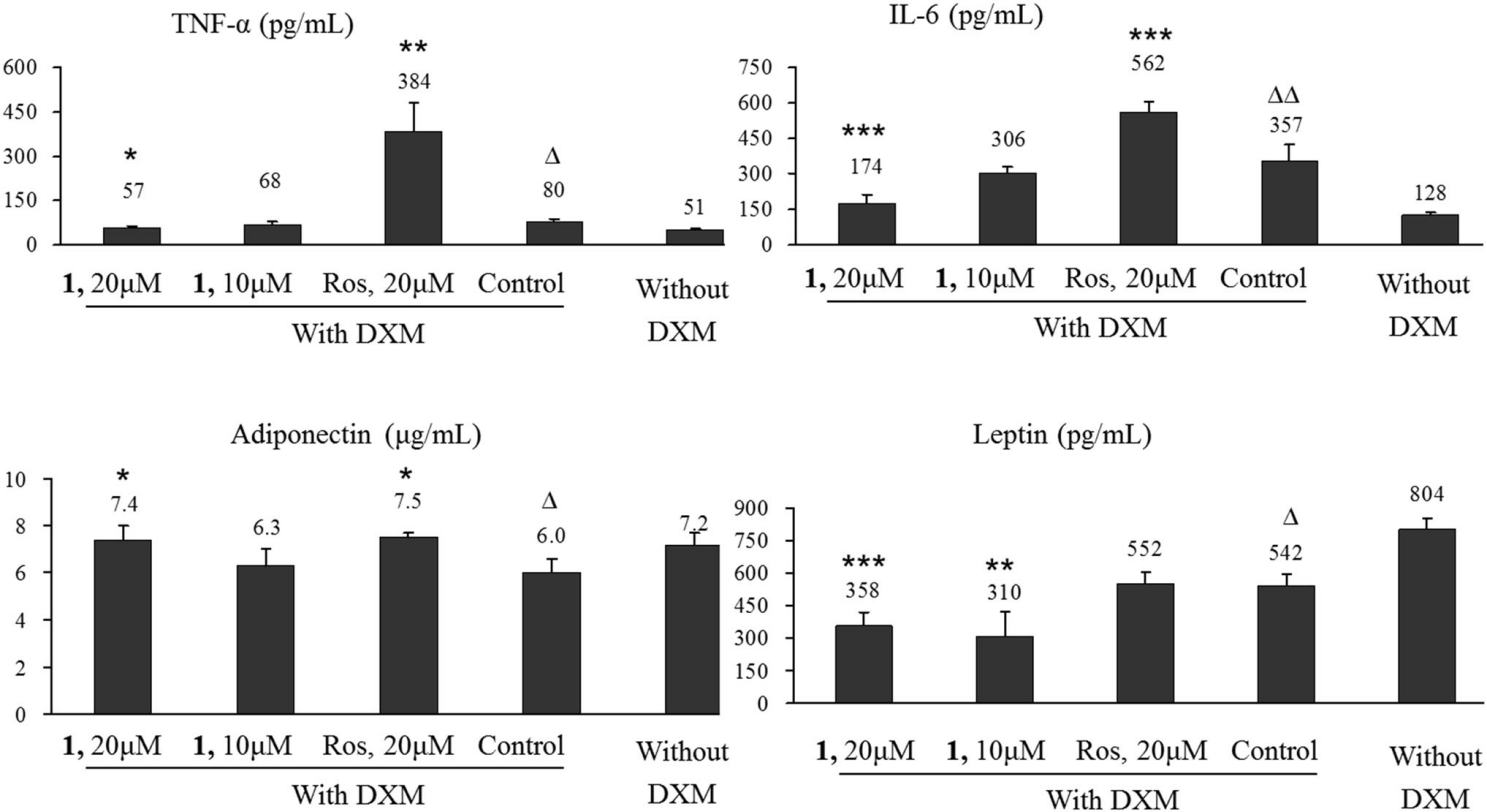
Compound **1** regulated cytokine secretion in DXM-treated 3T3-L1 adipocytes. Cells were incubated without 1 μM DXM together with 20 or 10 μM **1** or 20 μM rosiglitazone (Ros) for 48 h. Cytokine concentrations in culture medium were measured and expressed as mean ± SD of three independent experiments. ^Δ^ *P* < 0.05 and ^ΔΔ^ *P* < 0.01 versus DXM-free cells (Without DXM). ^*^ *P* < 0.05, ^**^ *P* < 0.01, and ^***^ *P* < 0.001 versus 3T3-L1 cells with DXM (Control)

Overexpressed proinflammatory cytokines (TNF-α, IL-6, leptin, etc.) and reduced expression of anti-inflammatory cytokine (adiponectin) are responsible for chronic inflammation and the development of insulin resistance [16]. It was reported that obese mice lacking either TNF-α or IL-6 show protection against developing insulin resistance, while chronic exposure to TNF-α, IL-6 or leptin activates PTP1B gene and reduces the expression of IRS proteins in myocytes or adipocytes of mice [17–19]. The anti-inflammatory cytokine adiponectin is specifically expressed by adipocyte and directly sensitizes the body to insulin [20]. Lack of adiponectin causes obesity and contributes to insulin resistance, type 2 diabetes, and the metabolic syndrome, while administration of adiponectin improves insulin resistance [21, 22]. Our study showed that, 3T3-L1 adipocytes in the presence of 1 μM DXM secreted more TNF-α (*P* < 0.05) and IL-6 (*P* < 0.01), but less leptin (*P* < 0.05) and adiponectin (*P* < 0.05) (Fig. 6) into medium than DXM-free ones, indicating severe disorder in cytokine secretion was induced by DXM treatment. Compound **1** at 20 μM significantly reduced the secretion of TNF-α (*P* < 0.05), IL-6 (*P* < 0.001) and leptin (*P* < 0.001), and elevated the secretion of adiponectin (*P* < 0.05) in DXM treated 3T3-L1 adipocytes, showing regulatory effect on DXM-induced adipocyte dysfunction (Fig. 6) that was different from rosiglitazone. Rosiglitazone at 20μM remarkably increased the secretion of TNF-α and IL-6 in 3T3-L1 adipocytes.

### Compound **1** Repaired Insulin Signaling Pathway and Activated PI3K/Akt2/AS160 Signaling in DXM- Induced Insulin Resistant 3T3-L1 Adipocytes

**Fig. 7:**
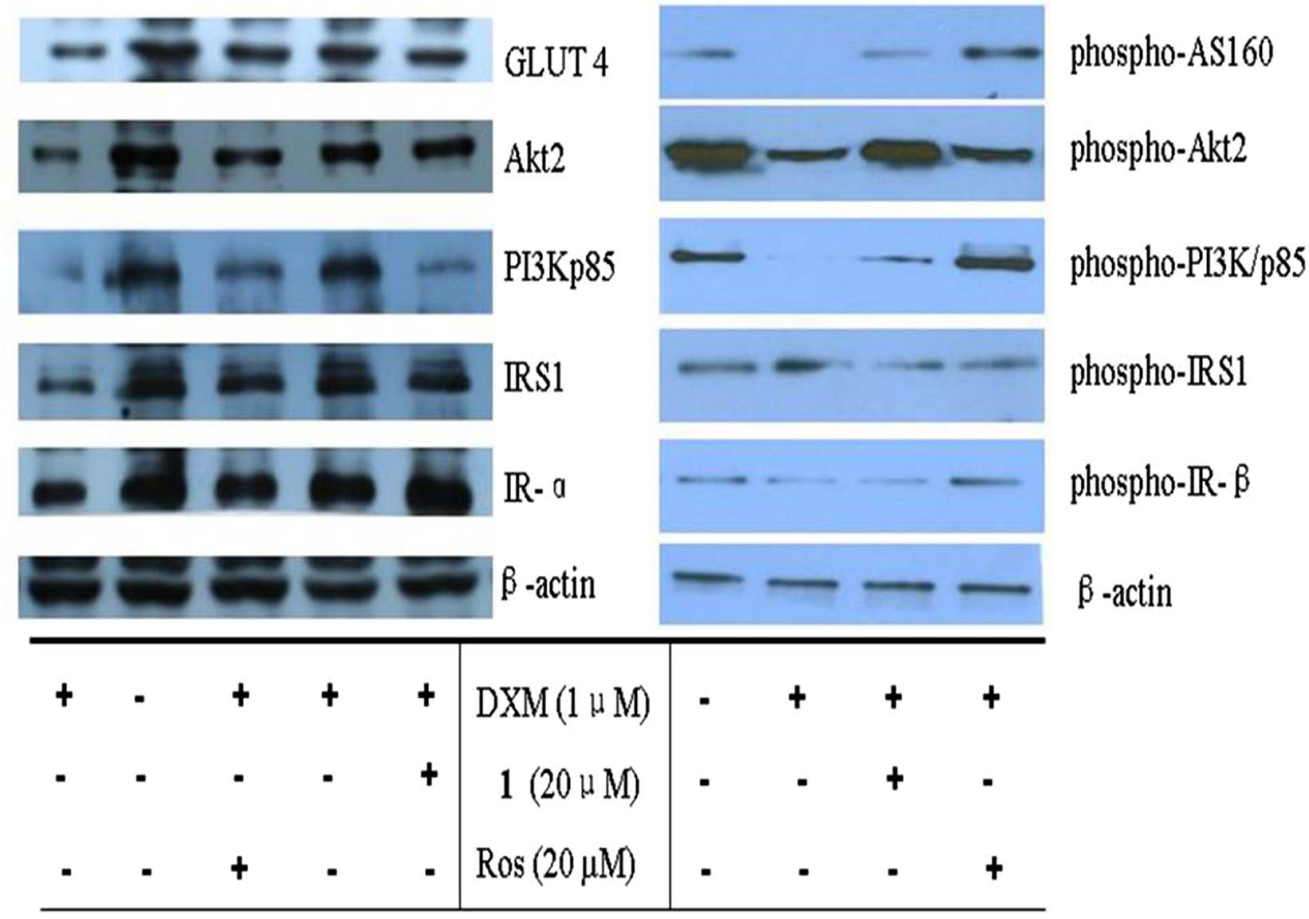
Compound **1** ameliorated DXM impaired insulin signaling pathway in DXM-3T3-L1 adipocytes. 3T3-L1 adipocytes were incubated with 1 μM DXM, with or without 20 μM **1** or 20 μM rosiglitazone (Ros) for 48 h. DXM-free 3T3-L1 adipocytes were set as insulin sensitive control. Protein expression was detected by western blotting

In mature adipocytes, glucose uptake is carried out by GLUT4 which is driven by insulin signaling transduction that is activated by sequential posphorylation of insulin signaling proteins, including IR, IRS1, PI3K and Akt2. Phosphorylated Akt2 then stimulates the translocation of intracellular GLUT4 vesicles to plasma membrane for functioning as glucose transporters [23]. Inhibition of signaling proteins leads to inhibited GLUT4 translocation and weakened glucose transporting ability of cells [24]. In which, the phosphorylation of down-stream PI3K/p85 and Akt2 plays a key role in GLUT4 translocation [25]. AS160, a substrate of Akt2, also regulates GLUT4 trafficking in adipocytes. Nonphosphorylated AS160 binds to intracellular GLUT4 vesicles and inhibits GLUT4 translocation, and AS160 phosphorylation overcomes this inhibitory effect [26]. Effects of 20 μM **1** on expression and/or phosphorylation of IR, IRS1, PI3K, Akt2, GLUT4 and AS160 in DXM-induced insulin resistant 3T3-L1 adipocytes were investigated. Result showed that, in DXM-treated 3T3-L1 adipocytes, expression of IR-α, IRS1, PI3K/p85, Akt2 and GLUT4 was suppressed (Fig. 7a), phosphorylation of down-stream proteins PI3K/p85, Akt2 and AS160 was obviously inhibited, while the phosphorylation of upper-stream proteins IR-β and IRS1 kept unchanged (Fig. 7b). The addition of 20 μM **1** to DXM-treated 3T3-L1 adipocytes elevated the expression of IR-α, IRS1, Akt2 and GLUT4 (Fig. 7a), and increased the phosphorylation of PI3K/p85, Akt2 and AS160, but did not affect the phosphorylation level of IR-β and IRS1, (Fig. 7b), showing activity similar to rosiglitazone. Result suggested that, DXM impaired insulin signaling pathway and reduced glucose consuming ability in 3T3-L1 adipocytes not by inhibiting upper-stream IR signaling transduction, but by directly inhibiting the down-stream PI3K/Akt2/AS160 signaling, thus leading to reduced GLUT4 translocation and glucose transporting ability. Compound **1** ameliorated DXM-induced impairment on insulin signaling pathway, and stimulated the phosphorylation of PI3K/Akt2/AS160, showing potential to deal with DXM-induced insulin resistance.

## Experimental Section

### Chemicals and Reagents

Cell counting kit-8 (CCK-8) was from Dojindo Molecular Technologies, Inc. (Kumamoto, Japan). Oil red O staining kit, FFA detection kit and TG assay kit were purchased from Nanjing Jiancheng Bioengineering Institute (Nanjing, China). Glycerol content GPO-POD enzymatic assay kit was purchased from Applygen Technologies Inc, China. ELISA kits for quantitative analysis of mouse TNF-α, IL-6, adiponectin, leptin and FAS, rabbit anti-IR-α, goat anti-rabbit IgG (γ-chain specific) and goat anti-mouse IgG (γ-chain specific) were from Wuhan Boster Biotech Co., Ltd (Wuhan, China). Insulin, 3-isobutyl-1-methylxanthine (IBMX), DXM and antibody for mouse GLUT4 were purchased from Sigma-Aldrich Chemical (St. Louis, MO, USA). Antibodies for IRS1, phospho-IRS1 (Ser307), PI3K/p85, phospho-PI3K/p85 (Tyr458)/p55 (Tyr199), Akt (pan), phospho-Akt2 (Ser473) and phospho-AS160 (Thr642) were purchased from CST China (Shanghai, China). Enhanced BCA Protein Assay Kit, Cell lysis buffer, PVDP membrane (0.45 μm), BeyoECL Plus were purchased from Beyontime Institute of Biotechnology (Shanghai, China). Rosiglitazone was provided by Guangzhou Institute for Drug Control (Guangzhou, China).

6α-Hydroxylup-20(29)-en-3-on-28-oic acid (**1**) was obtained from *Viburnum odoratissimum* in our laboratory [11]. It was dissolved in DMSO (20 mmol/L) and diluted with cell growth medium for experimental usage.

### Cell Line and Cell Culture

Murine preadipocyte cell line 3T3-L1 was kindly provided by Prof. WF Fong of Hong Kong Baptist University, and were grown in Dulbecco’s Modified Eagle’s Medium containing 4.5 g/L of glucose and 10 % fetal bovine serum (Gibco, Rockville, MD, USA), at 37 °C, saturate humidity and 5 % CO_2_.

### Cell Proliferation Assay

3T3-L1 preadipocytes in 100 μL growth medium were seeded in 96-well plates at a density of 5000 cells per well. Twenty-four hours later, different concentrations of **1** were added to the medium. After 48 h of incubation, cell viability was measured by using CCK-8 assay kit.

### Induction for Adipocyte Differentiation

3T3-L1 preadipocytes were induced for adipocyte differentiation by the “hormone cocktail” [11]. Briefly, the 4th generation cells were grown in growth medium until confluence. Cells were induced for adipocyte differentiation in growth medium containing 0.5 mM IBMX, 1 μM DXM and 10 μg/mL insulin for 2 days, and in growth medium containing 10 μg/mL insulin for another 2 days, and then in drug free growth medium for 4 days. Medium was replaced every 2 days. To investigate the effect of **1** on “hormone cocktail”-induced adipocyte differentiation, **1** of 20, 10 or 5 μM, or rosiglitazone of 20 μM was added together with the “hormone cocktail” on days 1–4.

### Detection of Adipocytes

Differentiated adipocytes were detected by staining the cellular lipid red with Oil red O [27]. After taking pictures under an inverted microscope, cellular Oil red O was dissolved with certain volume of isopropanol. Absorbance which is positively correlated to the rate of successful differentiation was read at 492 nm.

### Glucose Utilization, FFA and TG Synthesis in Adipocyte Differentiation

In 12-well plates, 3T3-L1 preadipocytes were induced for adipocyte differentiation for 8 days as described above, with 1.5 mL medium per well. **1** of various concentrations or rosiglitazone of 20 μM was added into the differentiation medium on days 1–4. Supernatant medium on days 6 was collected for measurement of glucose and FFA by using glucose assay kit and FFA detection kit, respectively. Cells on days 8 were washed with ice-cold PBS trice and then repeatedly frozen and thawed at −80/37 °C until the cells were fully disrupted. After addition of 100 uL of PBS, cells were shaved by Cell Scraper and the mixed liquor was collected and centrifuged for 5 min at 12.5 × 10^3^ g. Supernatant was collected for assessment of TG concentration by using the kit. Intracellular TG content was normalized to the concentration of total protein (TP) measured by using Enhanced BCA Protein Assay Kit.

### Measurement of Cellular FAS Content

In 6-well plates, 3T3-L1 preadipocytes were induced for adipocyte differentiation for 8 days as described above. Cells were washed with 2 mL PBS, lysed with 150 μL cell lysis buffer on ice for 2 min. Cell lysate was collected by centrifuge at 13.5 × 10^3^ g for 5 min at 4 °C. Content of FAS was measured by using the kit, intracellular FAS level was normalized to the content of total protein measured by using the protein assay kit.

### Glucose Consuming, Glycerol Releasing and Adipokine Secretion in DXM-Induced Insulin Resistant 3T3-L1 Adipocytes

In 6-well plates, fully differentiated 3T3-L1 adipocytes in 3 mL growth medium were incubated with 1 μM DXM for 48 h, with or without the addition of 20 or 10 μM of **1** or 20 μM of rosiglitazone. Supernatant medium was collected by centrifuge. Concentrations of glucose, glycerol, leptin, adiponectin, TNF-α and IL-6 were measured by using the kits.

### RT-PCR Analysis

One microgram of total RNA was subjected to RT reaction using Bestar qPCR RT Kit (DBI Bioscience, Ludwigshafen, Germany). RT reaction product was then amplified by PCR with the gene specific primers listed in Table 1 (Sangon Biotech, Shanghai, China) and Bestar^®^ SybrGreen qPCRmaster Mix (DBI) at 94 °C for 2 min, 94 °C for 20 s, 58 °C for 20 s, 72 °C for 20 s, and 40 cycles. The gene expression levels were analyzed by a StrataGene Mx3000P QPCR Real-Time PCR System (Agilent) and normalized using β-actin. Results were Mean ± SD from three independent experiments.

**Table 1:** Sequences of quantitative PCR primers for mouse genes

Gene name	Forward primer	Reverse primer
PPARγ	CAAGCTGAACCACCCAGAGT	CGATCTGCCTGAGGTCTGTC
C/EBPα	TGGAGGATTCCTGCTTCCTCT	TCTCAGCTTCCTGTATCTTCCT
β-Actin	CATTGCTGACAGGATGCAGA	CTGCTGGAAGGTGGACAGTGA

### Western-Blot Analysis

Thirty micrograms of total protein were separated in 10 % SDS–PAGE and electro-transferred to PVDF membranes. After blocking with 5 % BSA, membranes were incubated with primary antibodies in 2.5 % BSA, 0.3 % Tween-20 for 2 h at room temperature followed by corresponding secondary antibodies for another 1.5 h at room temperature. Protein bands were visualized by ECL.

### Statistical Analysis

All data are representative of three independent experiments. Values are expressed as mean ± SD. Statistical analyses were conducted using One-Way ANOVA. A value of *P* < 0.05 was considered to indicate statistical significance.

## Conclusion

6*α*-hydroxylup-20(29)-en-3-on-28-oic acid **(1**) inhibited hormone-induced adipocyte differentiation and lipogenesis in 3T3-L1 preadipocytes, alleviated DXM-induced adipocyte dysfunction and restored glucose consuming ability in DXM-treated 3T3-L1 adipocytes. It achieved this via down-regulating hormone-stimulated gene transcription of PPARγ and C/EBPα in 3T3-L1 preadipocytes, and via reactivating PI3K/Akt signaling and phosporylating AS160 in DXM-treated 3T3-L1 adipocytes, thus leading to increased GLUT4 translocation and enhanced glucose transportation.
